# Treatment discontinuation in pharmacological clinical trials for gambling disorder

**DOI:** 10.1016/j.jpsychires.2024.03.040

**Published:** 2024-03-25

**Authors:** Samuel R. Chamberlain, Konstantinos Ioannidis, Jon E. Grant

**Affiliations:** a Department of Psychiatry, Faculty of Medicine, University of Southampton, UK; NHS Southern Gambling Service / Southern Health NHS Foundation Trust, Southampton, UK; b Department of Psychiatry & Behavioral Neuroscience, University of Chicago, Pritzker School of Medicine, Chicago, IL, USA

**Keywords:** pharmacotherapy, discontinuation, treatment, gambling

## Abstract

**Background:**

Gambling disorder affects 0.5-2% of the population, and of those who receive treatment, dropout tends to be relatively high. Very little is known about participant-specific variables linked to treatment discontinuation/dropout in gambling disorder, especially in pharmacological clinical trial settings.

**Methods:**

Data were pooled from eight previous randomized, controlled pharmacological clinical trials conducted in people with gambling disorder who had never previously received any treatment for the disorder. Demographic and clinical variables were compared between those who did versus did not subsequently dropout from those treatment trials.

**Results:**

The sample comprised data from 635 individuals, and the overall rate of treatment dropout was 40%. Subsequent treatment dropout was significantly associated with the following: positive family history of gambling disorder in one or more first degree relatives (relative risk [RR] of dropout in those with positive history vs not = 1.30), preference for mainly strategic vs non-strategic gambling activities (RR= 1.43), lower levels of education (Cohen’s D=0.22), and higher levels of functional disability (Cohen’s D=0.18). These variables did not differ significantly as a function of treatment condition (medication versus placebo). Dropouts and completers did not differ significantly in terms of the other demographic or clinical variables that were considered.

**Conclusions:**

This study identified several candidate participant-specific predictors of pharmacological treatment dropout in gambling disorder. The findings highlight the need for future studies to address a wider range of contextual variables at large scale (including also study-specific variables e.g. trial/intervention duration), including in naturalistic treatment and clinical trial settings, with a view to developing algorithms that might usefully predict dropout risk.

## Introduction

Gambling disorder affects 0.4-2.4% of the population across the world and is linked to a variety of untoward outcomes such as high levels of comorbidities, disability, bankruptcy, and suicidality (Hodgins et al., 2011). Many people with gambling disorder do not receive treatment (e.g. due to stigma, lack of awareness, and lack of treatment facilities) (Braun et al., 2014), despite the fact that efficacious evidence-based treatment options exist. Examples of evidence-based treatments include cognitive behavioral therapy (CBT) (Pfund et al., 2023) ideally incorporating additional aspects such as motivational interviewing, and certain medications – such as the opioid antagonists, naltrexone and nalmefene (Hodgins et al., 2011; Ioannidis et al., 2023). With any treatment for a given mental disorder, a proportion of patients discontinue treatment. In gambling disorder, high dropout numbers (up to 66%) have been reported in pharmacological trials (Grant et al., 2006; Bartley & Bloch, 2013). In a meta-analysis of clinical trials of psychological treatment for gambling disorder, the average dropout rate was around 39.1% (95% confidence interval 33.0%-45.6%), which appears somewhat lower than that typically observed for medications (Pfund et al., 2021).

In a handful of treatment trials (medication/therapy) for alcohol use disorders, typical dropout rates have been reported to be 10-35%, and reasons for dropout remain largely unclear despite efforts to explore variables that may be important (Hallgren & Witkiewitz, 2013). Interestingly, dropout in clinical trials for people with co-occurring alcohol use disorder and bipolar disorder were found to be 40-74% in a small number of studies (Prisciandaro et al., 2011); and data from one of these trials linked risk-taking behaviors to higher risk of dropout (Prisciandaro et al., 2011). In a meta-analysis of antipsychotic medication clinical trials for psychosis, the typical dropout rate was around 48.1% for second generation antipsychotics, and 55.4% for classic antipsychotics (Kemmler et al., 2005). No particular variables, of those examined, were found to relate to dropout rate across studies (except for type of medication and in some cases – perhaps unsurprisingly – lack of efficacy in the active treatment arms).

Given what is known from studies in other mental health conditions, as well as the relatively high dropout seen in pharmacological clinical trials for gambling disorder, a pertinent question to address is ‘what variables are linked to treatment dropout in gambling disorder?’ In a retrospective review of 50 patients’ notes who attended outpatient clinics and received medication (plus were also offered psychotherapy) (Grant et al., 2004a), 48% discontinued treatment, and dropout was associated with lack of response to treatment and not having a supportive environment. In a systematic review, dropout ranged from 14%-50% for psychological treatments of gambling disorder (Melville et al., 2007). Ten studies were identified that reported on variables linked to dropout from psychological treatments, with sample sizes per study ranging from 21 to 232 participants. Variables potentially linked to dropout from psychological treatments included older age, employment status (being unemployed), life stressors, lack of social support, longer duration of symptoms, impulsivity, and presence of comorbidities (anxiety, or alcohol/substance use disorder). The authors noted that available research was limited and that the field was characterized by inconsistencies. While not examining treatment dropout per se, a later systematic review examined predictors of outcomes from psychological treatments in gambling disorder (Merkouris et al., 2016). The study noted some consistencies across the two systematic reviews, in terms of variables linked to subsequent outcomes from psychological treatments: gambling behaviors, alcohol use, employment status, and impulsive traits (Merkouris et al., 2016). In a large UK study of 846 patients with gambling disorder presenting to an NHS gambling clinic for CBT treatment, 27.4% dropped out prior to commencing treatment, and 17.4% dropped out during treatment (Ronzitti et al., 2017). Dropout during treatment was associated with being a smoker, having a family history of gambling, and higher scores on the Problem Gambling Severity Index (a measure of gambling severity over the previous 12 months). In the earlier mentioned Pfund et al. (2021) meta-analysis of clinical trials using psychological treatments for gambling disorder, meta-regression found that being unmarried was linked to higher dropout across the studies. Several other variables were not significant in the meta-regression, such as age, sex, employment, symptom severity, or number of sessions (Pfund et al., 2021).

Overall, the available literature sheds some light on variables linked to discontinuation from psychological treatments for gambling disorder, albeit with inconsistent results. Very little is known, however, about variables linked to treatment discontinuation for pharmacological treatments in this condition. Such information may be useful in order to (1) try to reduce risk of dropout for patients who receive medication treatments (be that in clinical trial or naturalistic settings); and/or (2) enroll participants most likely to benefit from completion of treatment. Therefore, we aggregated data from eight previous pharmacological clinical trials for gambling disorder and investigated baseline participant-specific variables (e.g. demographics, clinical variables) linked to subsequent treatment discontinuation (dropout from those trials). We hypothesized that dropout would be associated with the following baseline variables: lower levels of education, being single, worse gambling symptoms, more comorbidities, smoking, and family history of gambling disorder.

## Material and Methods

### Subjects

Data were aggregated from participants who attended clinical trials at the University of Chicago and the University of Minnesota, USA, led by one of the authors (JEG) (Kim et al., 2001; Kim et al., 2002; Grant et al., 2003; Grant et al., 2006; Grant et al., 2007; Grant et al., 2008; Grant et al., 2010; Grant et al. 2014). Studies were all randomized controlled trials from 8 weeks to 16 weeks in duration and each utilized one of four different pharmacological interventions: naltrexone, nalmefene, N-acetyl-cysteine and paroxetine. The choice of trials included in this analysis was based on convenience i.e. data availability to the research team. All diagnoses of gambling disorder were made by an experienced board-certified psychiatrist, using the criteria set forth by the 4^th^ Edition of the Diagnostic and Statistical Manual (DSM-IV) (American Psychiatric Association, APA, 1994) and the diagnoses were later confirmed to be consistent with the current requirements for gambling disorder using the DSM-5 criteria (American Psychiatric Association, APA, 2013). Diagnoses were made using a validated instrument (see later).

The exclusionary criteria for these studies were: history of psychotic or bipolar disorder, any current psychotherapy, any current (past 3 months) illicit drug use, or inability to provide informed consent. Data from eight, randomized double-blind, placebo-controlled published trials were included (Kim et al., 2001; Kim et al., 2002; Grant et al. 2003; Grant et al., 2006; Grant et al., 2007; Grant et al., 2008; Grant et al., 2010; Grant et al., 2014).

All study procedures were carried out in accordance with the Declaration of Helsinki. The Institutional Review Boards of the University of Minnesota and/or of the University of Chicago approved the procedures and the accompanying consent forms for each of the studies. For each of the studies, after all procedures were explained, all participants provided informed written consent. Each study was carried out in accordance with the latest version of the Declaration of Helsinki.

### Assessments

Dropout was defined as an individual who enrolled in a clinical trial and started treatment but did not complete the course of treatment as specified in the protocol. For example, dropout would thus refer to people lost-to-follow-up, as well as those opting out due to side effects or any other reasons. Dropout status was determined by the chief investigator and a consistent operational definition was used across all the studies. A semi-structured rater-administered questionnaire was used to collect detailed information on demographic and clinical features of gambling (e.g., preferred types of gambling, amount of money lost, problems related to gambling). All participants included in these trials were drawn from settings where multiple types of gambling (i.e., both strategic and non-strategic) were available. To determine preferred form of gambling, participants were asked as part of the semi-structured clinical interview, which form of gambling they preferred. Strategic gambling was defined as games (e.g., cards, sports, and dog/horse-race wagering) in which skill or knowledge may have some impact on outcomes (Petry, 2003). Other games such as slots, lottery, and pull tabs, require no skill, and consequently, these were categorized as ‘non-strategic' gambling.

We undertook the family history method where the proband is asked about psychiatric and substance use problems in their first-degree relatives, despite its methodological limitations (Andreasen et al., 1977), as this method aligns most closely with how family history is evaluated clinically. When a participant was unsure of a diagnosis, it was not included.

In addition, participants completed the following instruments:

- Structured Clinical Interview for Gambling Disorder (SCI-GD) (Grant et al., 2004b) for diagnosis of gambling disorder. Clinician administered.
- Structured Clinical Interview for DSM-IV Axis I Disorders (SCID-I) (First et al., 1995) to identify mainstream psychiatric comorbidities. Clinician administered.
- Yale-Brown Obsessive-Compulsive Scale modified for Pathological Gambling (PG-YBOCS) to quantify symptom severity over the past seven days (Pallanti et al., 2005). The PG-YBOCS is clinician administered.
- Gambling Symptom Assessment Scale (GSAS) to measure overall self-reported symptom severity for the past week (Kim et al., 2009).
- Hamilton Depression Rating Scale (HAM-D) to measure severity of depressive symptoms (Hamilton, 1960).
- Hamilton Anxiety Rating Scale (HAM-A) to measure severity of anxiety symptoms (Hamilton, 1959).
- Sheehan Disability Scale (SDS) to measure overall disability / functioning (Sheehan, 1983).

### Data Analysis

The baseline demographic and clinical features of those who did and those who did not subsequently complete treatment in the clinical trials were compared using analysis of variance (ANOVA) for continuous variables or likelihood ratio chi-squares for categorical variables. For any variables found to be significantly associated with treatment dropout, we examined whether the given variable differed as a function of treatment status (active versus placebo) using an ANOVA, or a likelihood ratio chi-square test as appropriate. To provide a context of likely magnitude/importance of any variables that differed significantly between groups, we also provided Cohen’s D or Relative Risk (RR) as appropriate. This being an exploratory study where we wished to avoid risk of falsely assuming a variable was not important when it was (i.e. to minimize likelihood of false negatives), statistical significance was defined as p<0.05. This approach was also taken due to the sample size which meant that the study would not have been sufficiently powered to detect small effects of interest if correction for multiple comparisons had been undertaken.

## Results

Of the 635 participants in clinical trials, 253 failed to complete the trials thus yielding an overall rate of dropout from the pooled clinical trials of 40%. The baseline demographic characteristics of those who did not versus those who did complete treatment are provided in Table 1. Treatment dropout was associated with lower levels of education, of relatively small effect size (Cohen’s D=0.22). Education level did not significantly differ as a function of treatment status (active versus placebo) (F=0.891, p=0.411). It can be seen that the treatment non-completers and completers did not differ significantly on any of the other variables that were considered (all p>0.05).

Table 2 shows the baseline clinical characteristics relating to gambling in each of the two groups. Treatment non-completers had significantly higher rates of strategic gambling and significantly higher likelihood of a family history of gambling disorder in first-degree relatives, as compared to the treatment completers. The Relative Risk (RR) of treatment dropout in those with mainly strategic vs mainly non-strategic gambling was 1.30 (95% confidence interval [CI] 1.05-1.61). The relative risk (RR) of dropping out from the subsequent clinical trial in those with a positive family history of gambling disorder, versus no such history, was 1.43 (95% CI 1.09-1.87). Likelihood of strategic gambling did not differ as a function of treatment status (active versus placebo) (likelihood ratio chi-square=3.405, p=0.493). Likelihood of having a family history of gambling disorder did not differ as a function of treatment status (active versus placebo) (likelihood ratio chi-square=2.664, p=0.264). The two groups did not differ significantly in terms of amount lost to gambling in the past year, gambling severity, or likelihood of having received at least one previous treatment for gambling disorder. The groups also did not differ in terms of average age when they first started gambling, nor duration of untreated illness.

The baseline clinical features of the two groups for the other variables are shown in Table 3. The treatment non-completers exhibited significantly higher levels of disability on the Sheehan Disability Scale (SDS) than the treatment completers, of relatively small effect size (Cohen’s D=0.18). Disability did not differ significantly as a function of treatment status (active versus placebo) (F=1.567, p=0.210). The groups did not differ significantly in terms of rates of mental health comorbidities, severity of anxiety/depression, or history of alcohol use disorder (whether in the probands or in their first-degree relatives).

In those who discontinued treatment, we conducted a post hoc analysis using a Cox’s proportional hazards model to examine whether variables linked to dropout could account for variation in time of dropout (i.e. weeks completed). The model was not significant (chi-square=2.902, p=0.715).

## Discussion

This study explored baseline variables associated with subsequent treatment dropout in a relatively large sample (N=635) of people with gambling disorder who participated in randomized, controlled pharmacological clinical trials. The overall dropout was approximately 40%. Our hypotheses were partly confirmed. Overall, treatment dropout was significantly associated with a positive family history of gambling disorder, strategic types of gambling, lower levels of education, and higher levels of psychosocial disability. Contrary to some of our expectations (which were based largely on the psychological treatment literature due to a lack of direct literature on medications), we did not observe any significant differences between completers and non-completers in terms of age, relationship status, smoking status, gambling symptom severity, or psychiatric comorbidities.

The overall rate of treatment discontinuation of around 40% with pharmacotherapies is by and large in keeping with figures typically reported in the literature for a variety of psychological treatments for gambling disorder (Ronzitti et al., 2017; Melville et al., 2007). Ronzitti et al. report 44.8% overall (27.4% pre-treatment and 17.4% during), while Melville et al. report 31% overall. To put this in an addiction context, this finding is also similar to that found in participants with substance use disorders who entered clinical trials using pharmacotherapy (40.4%) (Kranzler et al., 1996). The novel aspect is this is the first time this figure has been calculated from an aggregate set of pharmacological treatment trials for gambling disorder, as opposed to being reported in single trials. Such approach can provide more generalizable and statistically robust insights into the causes of treatment drop out from pharmacological interventions in gambling disorder.

What might account for the link between family history of gambling disorder and higher likelihood of treatment discontinuation? Ronzitti et al. 2017 also found a similar association between family history of gambling and in-treatment dropout. One potential explanation is that family history could be a proxy variable for the home and/or family environment: if gambling is relatively normalized and others are doing it in a person’s close family, then the affected individual may find it more difficult to consistently engage with a treatment program – whether due to relapse of symptoms, and/or experiencing particular permissive attitudes towards gambling. Similarly, there is some research suggesting that the participant may adopt views regarding the potentially positive financial and mood effects of gambling from their parents (Dowling et al., 2018). In clinical treatment trials, those views may contribute to treatment pessimism and enhance the chances of disengagement from treatment. The other finding that psychosocial dysfunction is also related to treatment discontinuation may further be linked to the home and/or family environment. Higher overall functional disability would be expected to make it harder to engage consistently with treatment in general, due to the involving nature of clinical trials and duration of the commitment.

The link between strategic gambling and higher likelihood of treatment dropout is intriguing. In a previous study examining personality traits in treatment-seeking people with gambling disorder, strategic gambling was associated with higher lack of perseverance, which in turn was linked to higher treatment discontinuation (Mallorqui-Bague et al., 2019). Thus, a plausible explanation of this association between strategic gambling and dropout in the current dataset may be a propensity for higher lack of perseverance (i.e. a type of impulsivity leading to greater propensity to disengage) in strategic gamblers, which in turn would be expected to lead to higher treatment discontinuation from dropout.

Lower levels of education (in terms of the level of education obtained) were found to be linked to higher dropout in the current study. This has been reported previously for a number of mental health disorders including in some gambling studies, albeit the main focus has been on psychotherapy rather than medications (e.g. Palomäki et al., 2023). People with relatively lower levels of education may be at risk of being disadvantaged when it comes to participation in clinical trials and also at risk of being excluded or dropping out from treatments per se.

While this is one of the first studies to explore variables associated with treatment discontinuation in pharmacological clinical trials for gambling disorder, several limitations should be considered. Findings may not generalize to other settings or types of treatment – such as people who have received previous treatments. We only considered dropout versus non-dropout rather than more nuanced categories. Furthermore, because this was an aggregate pooling of historical trials, we did not collect all variables likely to be relevant to explaining variation in dropout. Future work may thus wish to include a broader range of measures – for example, variables from the latest developmental models of gambling disorder (Blanco et al., 2015), barriers to care and treatment-seeking, impulsive and compulsive tendencies, cognitive tasks (e.g. related to decision-making) (Quinn et al., 2023), and social circumstances such as the nature of the home and family environments and family norms. Although a number of significant associations were found, these were of relatively small effect size. This is to be expected given the multitude of variables, measured and unmeasured, likely to contribute to clinical trial dropout. Put differently, we had not expected to detect variables with a large effect size, since we would not have expected this to be realistic in terms of complexity of the disease. We did not correct for multiple comparisons since we were interested in detecting significant associations of small effect size, and the study would not have been sufficiently powered to do so had we done this. Consequently, the findings require replication ideally in larger sample sizes; it would also be valuable for future work to conduct meta-analyses when the number and nature of studies permit this to be done. Lastly, we examined participant-specific variables linked to dropout from pharmacological clinical trials in general (across a number of studies): future work could consider if specific types of medication are linked to different participant-specific reasons for dropout. It would also be important to explore study-specific (as opposed to participant-specific) variables linked to dropout in future work (for example, duration of clinical trials) – this was not examined in the current work (given that it included a relatively small number of studies).

In conclusion we found that dropout from pharmacological clinical trials in gambling disorder was associated with pre-treatment family history of gambling (specifically in first-degree relatives), preference for strategic gambling, lower levels of education, and higher levels of functional disability. Future work is now needed to capture predictive measures, using harmonized measurement sets, across both clinical trial and naturalistic settings for treatment of gambling disorder. It is hoped that if common measurements can be collected across investigators and organizations, eventually it may be possible to generate useful evidence-based algorithms to predict outcomes and improve treatment pathways.

## Figures and Tables

**Table 1:** Demographic characteristics of the treatment non-completer and completer groups.

	Treatment Completer status						
	Non-completer (N=253)		Completer (N=382)				
	Mean / N	Std Dev / %	Mean / N	Std Dev / %	F	P	Sig.
**Age, years**	45.68	12.43	47.43	11.49	3.339	0.0681	
**Sex**					0.205 LR	0.6507	
Female	127	50.00%	184	48.17%			
Male	127	50.00%	198	51.83%			
**Racial-ethnic group**					4.769 LR	0.4447	
Caucasian	198	80.49%	305	86.16%			
African American	24	9.76%	28	7.91%			
Latino/Hispanic	13	5.28%	9	2.54%			
Asian	6	2.44%	6	1.69%			
Native American	4	1.63%	4	1.13%			
Other	1	0.41%	2	0.56%			
**Education level**	2.94	1.04	3.16	0.98	4.3115	0.0385	*
**Relationship status**					4.572 LR	0.470	
Single	45	30.61%	76	29.69%			
Not single	102	69.4%	180	70.3%			
**Height, inches**	67.64	4.21	67.76	3.71	0.0909	0.7632	
**Weight, pounds**	199.15	125.48	193.50	45.81	0.5579	0.4554	
**Smoker?**					0.004	0.9495	
No	69	46.94%	121	47.27%			
Yes	78	53.06%	135	52.73%			

Statistical tests are analysis of variance except where indicated LR = Likelihood ratio chi-square test. Education level is a score reflecting the highest level of education obtained to date, ranging from 0 (did not complete initial basic schooling) through to 5 (higher degree completed). For relationship data, presented as single vs not for simplicity for analysis carried out for full categories (e.g. single, married, cohabiting, etc.) Note that total cell sizes per group may differ due to missing data for some variables.

* p<0.05.

**Table 2:** Clinical characteristics related to gambling for the treatment non-completer and completer groups.

	Treatment Completer status						
	Non-completer (N=253)		Completer (N=382)				
	Mean / N	Std Dev / %	Mean / N	Std Dev / %	F	P	Sig.
**Dollars (USD) lost to gambling in the past year**	26203	31157	25054	33952	0.0874	0.7677	
**GSAS**	35.29	11.20	33.70	9.51	3.5986	0.0583	
**PG-YBOCS**	23.45	5.52	22.89	4.69	1.5605	0.2122	
**Age when first started to gamble, years**	25.24	13.34	26.60	13.03	1.4505	0.229	
**Duration of Untreated Illness, years**	9.78	8.47	10.08	8.30	0.1812	0.6705	
**Strategic or non-strategic gambler (of those who show preference)**					5.765 LR	0.0163	*
Strategic	96	48.24%	113	37.42%			
Non-strategic	103	51.76%	189	62.58%			
**Previous Gambling Treatment**					2.422	0.1196	
No	130	60.75%	166	53.90%			
Yes	84	39.25%	142	46.10%			
**Family history of gambling disorder (1’ relative)**					7.116 LR	0.0076	**
No	60	41.96%	143	55.86%			
Yes	83	58.04%	113	44.14%			

Statistical tests are analysis of variance except where indicted LR = Likelihood ratio chi square test. GSAS = Gambling Symptom Assessment Scale; PG-YBOCS = Yale-Brown Obsessive-Compulsive Scale Modified for Pathological Gambling. For strategic vs non-strategic, this analysis focused on those who showed a preference for one or the other. Note that total cell sizes per group may differ due to missing data for some variables.

* p<0.05

** p<0.01.

**Table 3:** Other clinical characteristics in the treatment non-completer and completer groups.

	Treatment Completer status						
	Non-completer (N=253)		Completer (N=382)				
	Mean / N	Std Dev / %	Mean / N	Std Dev / %	F	p	Sig.
**Number of current comorbidities (mainstream mental disorders)**					8.149 LR	0.0863	
0	153	72.51%	192	65.08%			
1	40	18.96%	71	24.07%			
2	13	6.16%	29	9.83%			
3	5	2.37%	2	0.68%			
4	0	0.00%	1	0.34%			
**Lifetime alcohol use disorder**					0.669 LR	0.4133	
No	184	82.14%	289	84.75%			
Yes	40	17.86%	52	15.25%			
**HAMA**	7.76	4.53	7.30	4.68	0.908	0.341	
**HAMD**	7.64	4.27	7.01	4.06	2.072	0.151	
**Sheehan Disability Scale**	16.65	6.81	15.44	6.55	3.914	0.049	*
**Family history of alcohol use disorder (1’ relative)**					0.116 LR	0.734	
No	67	46.85%	124	48.63%			
Yes	76	53.15%	131	51.37%			

Statistical tests are analysis of variance except where indicated LR = Likelihood ratio chi square test. HAMA = Hamilton Anxiety Rating Scale; HAMD = Hamilton Depression Rating Scale. Note that total cell sizes per group may differ due to missing data for some variables.

* p<0.05.
